# Synergistic Effects of Selenium and Silicon Nanoparticles on Peach Quality Enhancement and Se Biofortification Through Foliar Application

**DOI:** 10.3390/plants14233669

**Published:** 2025-12-02

**Authors:** Ziyang Wang, Bilal Hussain, Xin Wang, Yasir Hamid, Jiali Zhang, Muhammad Bilal Khan, Zhenli He, Xiaoe Yang

**Affiliations:** 1 National Health Agriculture Industry Research and Development Center, Zhejiang Green & Health ECO-Agriculture LLC, Zhejiang University Alumni Headquarters Economy Park, No 396, Unit A4-1 Cangxing Street, Yuhang District, Hangzhou 311121, China; yangyang1554@gmail.com; 2 Ministry of Education (MOE) Key Laboratory of Environmental Remediation and Ecosystem Health, College of Environmental and Resources Science, Zhejiang University, Hangzhou 310058, China; 12414011@zju.edu.cn (X.W.); yasirses2007@gmail.com (Y.H.); zjli557@163.com (J.Z.); 3 Indian River Research and Education Center, Institute of Food and Agricultural Sciences, University of Florida, Fort Pierce, FL 34945, USA; mb.khan@ufl.edu (M.B.K.); zhe@ufl.edu (Z.H.)

**Keywords:** Se biofortification, bio-accessibility, peach quality, foliar application, antioxidant enhancement

## Abstract

Selenium (Se) biofortification represents a critical strategy for addressing micronutrient deficiency while enhancing fruit nutritional quality. This study investigated foliar applications of Se and Si nanoparticles (NPs) for peach Se biofortification and quality enhancement. Se NPs (95.2 nm) were synthesized and characterized using SEM, EDS, and FTIR analyses. Six treatments were applied: control (Ck), SeNPs-5, SeNPs-10, SiNPs-10, Se5Si10, and Se10Si10. SeNPs-10 achieved maximum Se biofortification (0.47 mg kg^−1^), representing 5.4-fold increases over controls, with 85% organic Se accumulation. Combined treatments demonstrated synergistic effects on multiple quality parameters. Se5Si10 led to the highest antioxidant enzyme activities (peroxidase: 2254 U g^−1^, catalase: 61.7 U g^−1^) and phenolic compound enhancement (chlorogenic acid: 267 mg kg^−1^, total phenolics: 12.8 mg GAE g^−1^). Flavonoid biosynthesis was optimized with Se10Si10 achieving maximum rutin accumulation (53.9 mg kg^−1^) and ascorbic acid content (60.7 mg/100 g). Physical quality improvements included enhanced firmness (100.9 N cm^−2^) and sugar accumulation (14.1% soluble solids). Combined treatments reduced oxidative stress markers (MDA: 22.11 μmol g^−1^) while enhancing protein metabolism. These findings demonstrate that Se-Si nanoparticle combinations showed optimal biofortification with synergistic quality enhancement, establishing effective strategies for nutritionally enriched peach production.

## 1. Introduction

Se deficiency represents a critical global health challenge affecting an estimated 1 billion people worldwide, with particularly severe implications for populations in Se-deficient regions including vast areas of China, sub-Saharan Africa, and parts of Europe [1,2], while in China, approximately 72% of the territory exhibits Se-deficient soils with concentrations below 0.125 mg kg^−1^, directly impacting food chain Se levels and human dietary intake [3,4]. This widespread deficiency contributes to increased susceptibility to oxidative stress-related diseases, compromised immune function, and reduced antioxidant capacity in affected populations [5,6]. The WHO recognizes Se as an essential trace element crucial for human health, with recommended daily intakes of 55 μg for adults, yet dietary surveys consistently demonstrate inadequate Se consumption in Se-deficient regions [7].

The physiological importance of Se arises from its incorporation into selenoproteins, including glutathione peroxidases, thioredoxin reductases, and selenoprotein P, which serve critical roles in antioxidant defense, thyroid hormone metabolism, and immune system regulation [8]. Se deficiency has been linked to increased cancer risk, cardiovascular disease, cognitive decline, and viral susceptibility, highlighting the urgent need for effective biofortification strategies [4]. Traditional approaches to address Se deficiency through dietary supplements face challenges related to cost, accessibility, and consumer compliance, emphasizing the importance of food-based biofortification as a sustainable solution [9].

Peach (*Prunus persica*) production represents a significant economic sector globally, with China contributing approximately 60% of world production at 15.2 million tons annually (FAO, [10]). Beyond economic importance, peaches serve as valuable dietary sources of bioactive compounds including phenolic acids, flavonoids, carotenoids, and vitamin C, which contribute to their recognized health-promoting properties [11]. Consumer demand increasingly emphasizes fruit quality attributes encompassing not only traditional parameters such as sugar content, firmness, and shelf-life, but also nutritional density and functional compound concentrations [12]. The integration of nutritional biofortification with quality enhancement represents a promising approach to develop premium fruit products that address both consumer preferences and public health objectives.

Peach quality encompasses complex interactions between physical attributes, chemical composition, and sensory characteristics that determine market value and consumer acceptance [13]. Key quality determinants include fruit firmness, soluble solid content, titratable acidity, and aromatic compound profiles, which collectively influence texture, sweetness, flavor intensity, and storage potential [14]. Additionally, antioxidant capacity derived from phenolic compounds and flavonoids contributes significantly to nutritional quality and potential health benefits, with quercetin, chlorogenic acid, and catechin representing major bioactive constituents [15]. The challenge lies in simultaneously enhancing multiple quality parameters while achieving effective micronutrient biofortification without compromising horticultural performance.

Traditional Se supplementation approaches in agriculture have relied primarily on soil application of selenate or selenite fertilizers, which face limitations including uneven distribution, environmental leaching, and variable plant uptake efficiency [12]. Foliar application methods offer improved bioavailability but conventional Se salts may cause phytotoxicity at concentrations required for effective biofortification [16]. These limitations have driven research toward nanotechnology-based delivery systems that enhance nutrient use efficiency while minimizing environmental impact and plant stress.

Nanotechnology applications in agriculture have emerged as transformative approaches for precision nutrient delivery, offering advantages including enhanced bioavailability, controlled release, and reduced input requirements compared to conventional fertilization methods [17]. Se NPs demonstrate superior plant uptake efficiency and reduced toxicity compared to ionic forms, attributed to their unique physicochemical properties including high surface area-to-volume ratios and size-dependent cellular penetration mechanisms [18]. Recent advances in nanoparticle synthesis have enabled production of uniform, stable Se NPs with optimal size ranges for plant uptake, typically 20–100 nm, which exhibit enhanced biocompatibility and metabolic integration [19].

Silicon (Si), the second most abundant crustal element, enhances plant stress tolerance and metabolic function despite not being essential for all species [4]. Si enhances nutrient uptake efficiency by modulating cell wall porosity through pectin cross-linking and regulating aquaporin function, thereby facilitating systemic nutrient translocation [20]. Importantly, Si has been shown to upregulate sulfate transporter expression, including SULTR1;2 and SULTR2;1, which are also responsible for Se movement due to the chemical similarity between sulfate and selenate [21]. Furthermore, Si improves fruit quality attributes including sugar accumulation and phenolic compound biosynthesis through enhanced photosynthetic capacity and optimized cellular metabolism, even under favorable growing conditions [4].

Previous studies have demonstrated beneficial effects of Se and Si nanoparticles in several crops, including rice [1,4], wheat [22], cucumber [23], and strawberry [24], where improvements in nutrient uptake, stress tolerance, and antioxidant capacity were observed. However, to the best of our knowledge, no prior research has focused on the combined foliar application of Se and Si nanoparticles in peach (*Prunus persica*), particularly with respect to simultaneous Se biofortification and fruit quality enhancement. This knowledge gap highlights the novelty and importance of the present study. This dual nanoparticle system leverages complementary mechanisms: Se providing direct nutritional enhancement and antioxidant benefits, while Si optimizing cellular conditions for enhanced nutrient uptake, and quality development.

This study addressed the critical knowledge gap regarding synergistic effects of combined Se and Si nanoparticle applications on peach biofortification and quality enhancement. Our primary objective was to evaluate the effectiveness of foliar-applied Se and Si NPs for enhancing fruit Se content and simultaneously antioxidant capacity, phenolic compound accumulation, physical quality attributes. Combined Se-Si treatments would demonstrate synergistic effects on fruit quality through coordinated activation of antioxidant defense systems and secondary metabolite biosynthetic pathways.

## 2. Results

### 2.1. Se and Si Nanoparticles Characterization

**Figure 1:**
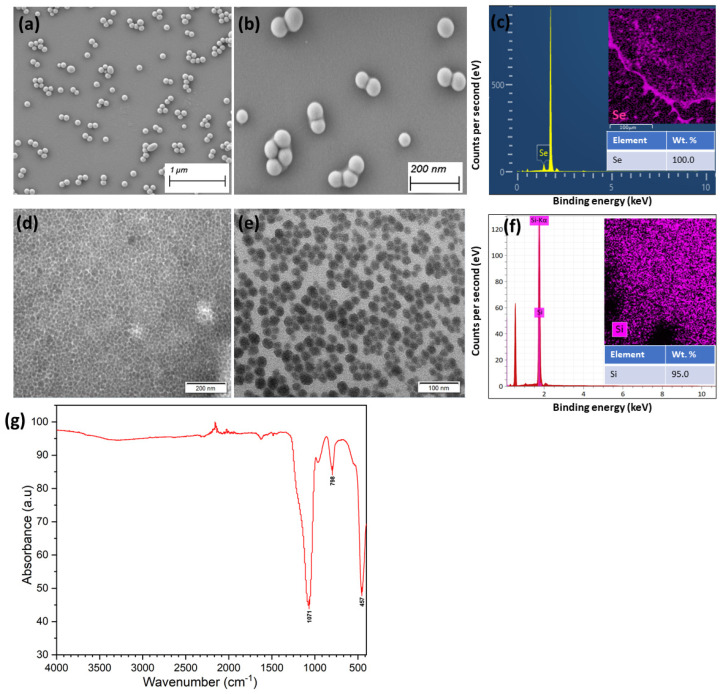
Characterization of Se- and Si-NPs. (**a**,**b**) SEM images of Se-NPs showing spherical shape and uniform size distribution (scale bars: 1 µm and 200 nm). (**c**) EDS spectrum and elemental mapping of Se-NPs (inset). (**d**,**e**) SEM images of Si-NPs showing uniform spherical morphology (scale bars: 200 nm and 100 nm). (**f**) EDS spectrum and elemental mapping of Si-NPs (inset). (**g**) FTIR spectrum showing characteristic absorption bands of Se-NPs.

The synthesized Se and commercially purchased Si NPs demonstrated exceptional uniformity and monodispersity as revealed through comprehensive physicochemical characterization. **SeNPs:** SEM analysis (Figure 1a) showed predominantly spherical Se NPs with uniform size distribution across the synthesis matrix, exhibiting particle sizes ranging from 70 to 110 nm with minimal aggregation tendencies. The SEM imaging (Figure 1b) confirmed the smooth surface morphology and consistent spherical geometry of individual NPs, with an average diameter of 95.2 ± 8.7 nm.

Energy-dispersive X-ray spectroscopy coupled with elemental mapping (Figure 1c) unequivocally confirmed the elemental composition, revealing 100% Se content with characteristic Se Lα peak at 1.379 keV and Se Kα peak at 11.22 keV. The homogeneous distribution of Se across the nanoparticle matrix, as evidenced by the purple-colored mapping overlay, indicated successful synthesis without elemental impurities or oxidation products.

**Si NPs:** SEM analysis (Figure 1d,e) revealed highly uniform Si NPs with excellent dispersion characteristics. The TEM images at different magnifications demonstrated spherical Si NPs with an average diameter of approximately 24 ± 4.3 nm, showing remarkable size homogeneity and minimal aggregation. The EDS analysis and elemental mapping (Figure 1f) confirmed the high purity of Si NPs, with the characteristic Si Kα peak and homogeneous Si distribution across the analyzed area (95.0 wt.% Si), validating highly pure Si nanostructures.

Fourier-transform infrared spectroscopy (Figure 1g) revealed the surface chemistry and stabilization mechanism of the Se NPs. The spectrum exhibited characteristic absorption bands at 1635 cm^−1^ corresponding to C=C stretching vibrations, and a prominent peak at 1384 cm^−1^ attributed to Se–O stretching, indicating partial surface oxidation that enhances nanoparticle stability. The absence of broad O–H stretching bands around 3200–3600 cm^−1^ confirmed minimal water adsorption, while the sharp peaks in the fingerprint region (800–1500 cm^−1^) demonstrated the crystalline nature of the synthesized Se nanostructures.

The integrated characterization data collectively confirmed the successful synthesis of highly pure, monodisperse spherical Se NPs with optimal size distribution and surface properties suitable for agricultural biofortification applications.

### 2.2. Fruit Yield and Si Accumulation

Foliar application of Se and Si nanoparticles did not significantly affect fruit yield parameters in peach trees (Table 1). Fruit yield ranged from 52.4 ± 3.8 kg/tree in the control to 55.8 ± 4.3 kg/tree in the SiNPs-10 treatment, with no statistically significant differences observed among treatments (*p* > 0.05). Similarly, the number of fruits per tree (312–335 fruits) and average fruit weight (167–169 g) remained consistent across all treatments, demonstrating that nanoparticle applications at the tested concentrations maintained normal crop productivity without any adverse effects.

Silicon content in shoots, however, showed significant treatment effects (*p* < 0.05). The SiNPs-10 treatment resulted in a 78% increase in shoot Si concentration (1.46 ± 0.09 mg g^−1^ DW) compared to the control (0.82 ± 0.06 mg g^−1^ DW). Combined Se-Si treatments also demonstrated significantly elevated Si levels, with Se5Si10 (1.38 ± 0.08 mg g^−1^ DW) and Se10Si10 (1.32 ± 0.07 mg g^−1^ DW) showing 68% and 61% increases over control, respectively. In contrast, selenium-only treatments (SeNPs-5 and SeNPs-10) did not affect Si accumulation, maintaining levels similar to the control. These results confirm successful foliar uptake and translocation of Si nanoparticles while demonstrating that the biofortification and quality enhancement effects observed in subsequent analyses were achieved without compromising fruit yield.

### 2.3. Se Concentration and Speciation

**Figure 2:**
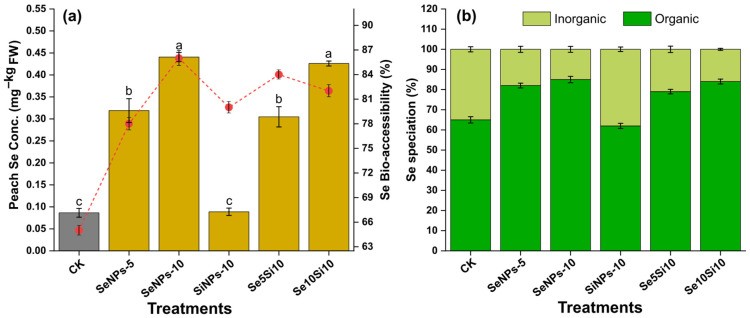
Se bioaccumulation and bio-accessibility in peach fruit following nanoparticle treatments. (**a**) Se concentration in peach fruits (bars, left y-axis) and Se bio-accessibility % (red line with circles, right y-axis) under different treatments. (**b**) Distribution of Se forms showing organic and inorganic fractions. Data represent means ± standard error of three biological replicates. Different letters indicate significant differences between treatments at *p* < 0.05.

The foliar application of Se nanoparticle treatments exhibited remarkable enhancement in fruit Se accumulation with distinct dose-dependent responses (Figure 2a). SeNPs-10 elicited the highest fruit Se concentration (0.47 mg kg^−1^ FW), representing a 5.4-fold increase over control (CK: 0.09 mg kg^−1^). The Se nanoparticle treatments SeNPs-5 (0.32 mg kg^−1^) led to intermediate bioaccumulation levels, while Si nanoparticle treatment SiNPs-10 (0.09 mg kg^−1^) showed the lowest Se enhancement. Combined treatments Se5Si10 (0.31 mg kg^−1^) and Se10Si10 (0.43 mg kg^−1^) maintained Se bioaccumulation levels similar to those of their respective individual Se treatments. Statistical analysis revealed no significant difference in Se bioaccumulation between the Se-NPs treatments and their corresponding combined Se + Si nanoparticle treatments (Se5Si10 vs. SeNPs-5, Se10Si10 vs. SeNPs-10).

Se bioaccessibility efficiency varied significantly between treatments, with SeNPs-10 exhibiting the highest bioaccessibility at 85% followed by combined treatments Se5Si10 (84.12%) and Se10Si10 (82.31%). The bioaccessibility pattern revealed optimal Se uptake under nanoparticle treatments, with SiNPs-10 showing limited Se accumulation (62% efficiency), confirming the specificity of Se nanoformulations for biofortification applications.

Analysis of Se speciation (Figure 2b) revealed preferential accumulation of organic Se forms with all Se-containing treatments. SeNPs-10 demonstrated high organic Se proportion (85%), while SeNPs-5 maintained 82% organic Se content, indicating efficient biotransformation of applied inorganic Se NPs into bioavailable organic seleno-compounds. The control treatment Ck showed predominantly organic Se baseline (65%), while combined treatments Se5Si10 (79%) and Se10Si10 (82%) maintained elevated organic Se ratios, confirming the highest bioconversion efficiency of Se nanoparticle formulations.

The integrated biofortification data collectively demonstrated that Se nanoparticle treatments, particularly SeNPs-10, achieved optimal fruit Se enrichment with predominantly organic Se accumulation, proving to be an effective biofortification strategy for enhancing the nutritional value of peach fruits.

### 2.4. Antioxidant Enzyme Activities and Total Antioxidant Capacity

**Figure 3:**
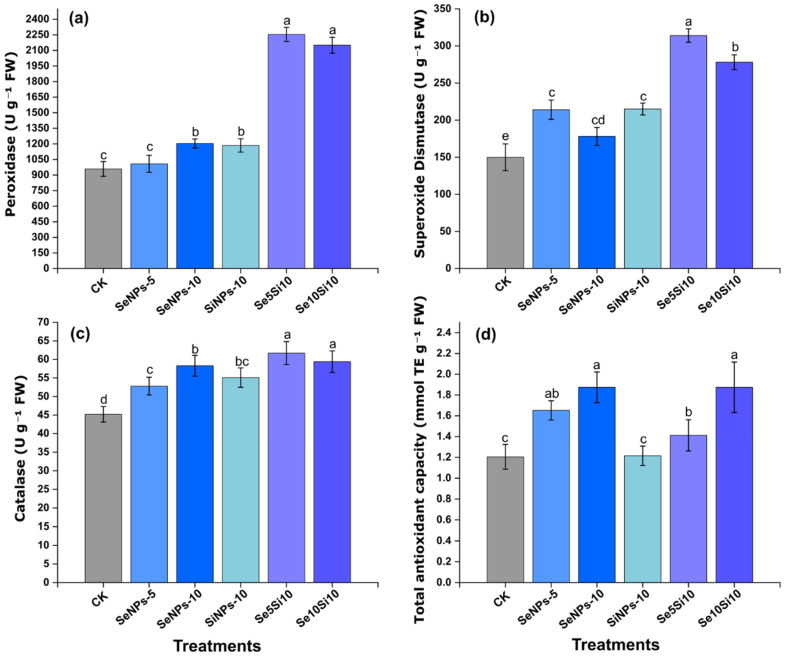
Effects of Se and Si nanoparticle treatments on antioxidant enzyme activities in peach fruit. (**a**) Peroxidase (POD), (**b**) Superoxide dismutase (SOD), (**c**) Catalase (CAT), and (**d**) Total antioxidant capacity. Data represent means ± standard error of three biological replicates. Different letters indicate significant differences between treatments at *p* < 0.05.

Se and Si nanoparticles treatments markedly enhanced the antioxidant defense system of peach fruits through coordinated enzyme activation (Figure 3). POD activity (Figure 3a) showed a strong treatment-dependent increase, with Se5Si10 achieving the highest activity (2254 U g^−1^ FW), followed by Se10Si10 (2150 U g^−1^ FW) both representing approximately 2.4-fold increases over the control (950 U g^−1^ FW). Individual treatments with SeNPs-5 (1008 U g^−1^) and SeNPs-10 (1204 U g^−1^) resulted in moderate enhancements, while SiNPs-10 (1185 U g^−1^) highlighted Si’s independent role in peroxidase activation.

SOD activity (Figure 3b) followed a similar trend. The combined treatments Se5Si10 (314 U g^−1^ FW) and Se10Si10 (278 U g^−1^ FW) exhibited the highest antioxidant capacities, surpassing those of the individual SeNPs-5 (214 U g^−1^) and SeNPs-10 (178 U g^−1^) treatments. The control treatment-maintained baseline SOD activity (150 U g^−1^). CAT activity (Figure 3c) further supported the synergistic effect of combined Se-Si applications. Se5Si10 (61.7 U g^−1^ FW) and Se10Si10 (59.4 U g^−1^ FW) showed maximal induction, whereas SeNPs-5 (52.8 U g^−1^) and SeNPs-10 (58.3 U g^−1^) produced moderate increases above the control (45.2 U g^−1^). Total antioxidant capacity (Figure 3d) reflected the cumulative impact of these enzymatic responses. SeNPs-10 and Se10Si10 both reached peak values (1.87 mmol TE g^−1^ FW), while Se5Si10 maintained a notably elevated level (1.41 mmol TE g^−1^) compared with the control (1.21 mmol TE g^−1^).

Overall, the coordinated activation of antioxidant enzymes demonstrates that combined Se-Si nanoparticle treatments effectively optimized the fruit’s cellular antioxidant defense system. Se5Si10 exhibited highest enzyme-specific stimulation, whereas Se10Si10 resulted in the second highest enhancement of total antioxidant capacity.

### 2.5. Phenolic Compound Profiles and Secondary Metabolite Enhancement

**Figure 4:**
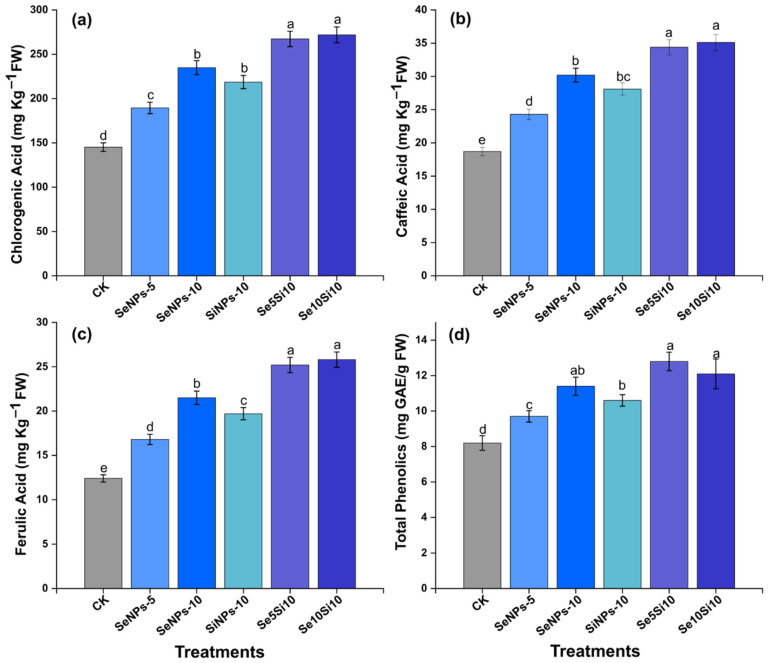
Effects of Se and Si nanoparticle treatments on phenolic acid profile in peach fruit. (**a**) Chlorogenic acid, (**b**) Caffeic acid, (**c**) Ferulic acid, and (**d**) Total phenolics content. Data represent means ± standard error of three biological replicates. Different letters indicate significant differences between treatments at *p* < 0.05.

Nanoparticle treatments significantly modulated peach fruit phenolic metabolism, enhanced bioactive compound accumulation across multiple phenolic classes (Figure 4). Chlorogenic acid content (Figure 4a) exhibited remarkable treatment-dependent increases with Se10Si10 achieving maximum accumulation (272 mg kg^−1^ FW), followed by Se5Si10 (267 mg kg^−1^ FW), representing 1.9-fold increases over control (145 mg kg^−1^). Individual Se treatments SeNPs-5 (189 mg kg^−1^) and SeNPs-10 (245 mg kg^−1^) demonstrated dose-dependent responses, while SiNPs-10 (219 mg kg^−1^) confirmed Si’s independent contribution to chlorogenic acid biosynthesis.

Caffeic acid biosynthesis (Figure 4b) displayed similar enhancement patterns with combined treatments Se5Si10 (34.4 mg kg^−1^ FW) and Se10Si10 (35.1 mg kg^−1^ FW) achieving highest accumulation compared to individual applications. SeNPs-10 (30.2 mg kg^−1^) showed elevated caffeic acid levels, while control-maintained baseline concentrations (18.7 mg kg^−1^). Ferulic acid content (Figure 4c) reinforced the synergistic effects of dual nanoparticle formulations with Se5Si10 (25.2 mg kg^−1^ FW) and Se10Si10 (25.8 mg kg^−1^ FW) demonstrating maximal ferulic acid induction. Individual treatments SeNPs-5 (16.8 mg kg^−1^) and SeNPs-10 (21.5 mg kg^−1^) achieved intermediate accumulation levels above control baseline (12.4 mg kg^−1^).

Total phenolic content (Figure 4d) integrated the cumulative phenolic enhancement effects with Se5Si10 (12.8 mg GAE g^−1^ FW) and Se10Si10 (12.1 mg GAE g^−1^ FW) achieving optimal phenolic enrichment. SeNPs-10 (11.4 mg GAE g^−1^) demonstrated substantial individual treatment efficacy, while control maintained lower phenolic levels (8.2 mg GAE g^−1^). The coordinated phenolic compound enhancement revealed that combined Se-Si nanoparticle treatments optimized secondary metabolite biosynthesis pathways, with Se5Si10 achieving highest individual phenolic acid accumulation and total phenolic enrichment under treatment applications.

### 2.6. Flavonoid Biosynthesis and Quality Marker Enhancement

**Figure 5:**
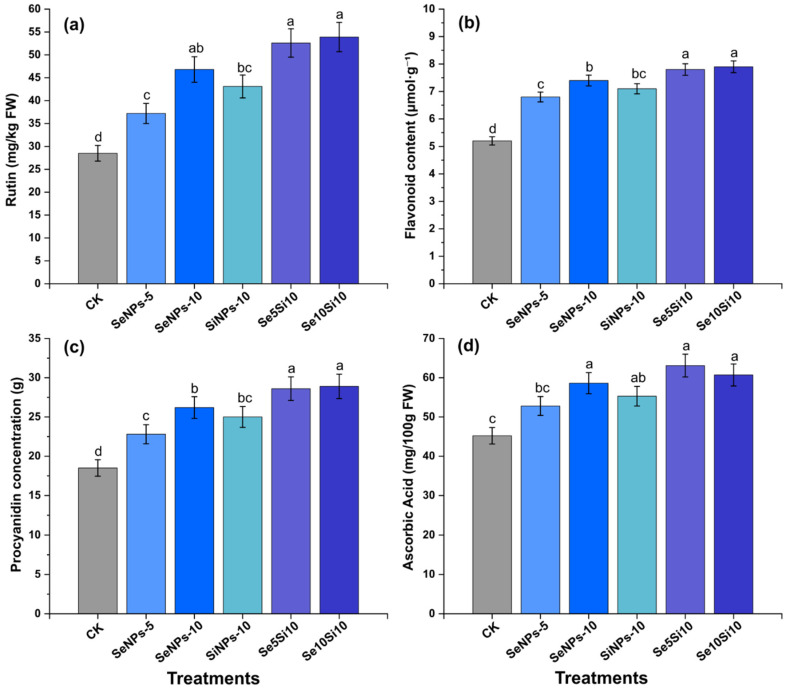
Effects of Se and Si nanoparticle treatments on flavonoids and antioxidant compounds in peach fruit. (**a**) Rutin content, (**b**) Flavonoid content, (**c**) Procyanidin concentration, and (**d**) Ascorbic acid content. Data represent means ± standard error of three biological replicates. Different letters indicate significant differences between treatments at *p* < 0.05.

Nanoparticle applications markedly enhanced flavonoid biosynthesis and the accumulation of quality-related metabolites in peach fruits (Figure 5). Rutin content (Figure 5a) showed significant treatment-dependent increases, with the combined treatments Se5Si10 (52.6 mg kg^−1^ FW) and Se10Si10 (53.9 mg kg^−1^ FW) achieving the highest flavonoid accumulation approximately 1.9-fold greater than the control (28.5 mg kg^−1^). Individual Se applications, SeNPs-5 (37.2 mg kg^−1^) and SeNPs-10 (46.8 mg kg^−1^), exhibited clear dose-dependent responses, while SiNPs-10 (43.1 mg kg^−1^) confirmed Si’s independent contribution to rutin biosynthesis.

Total flavonoid content (Figure 5b) further supported the synergistic enhancement pattern, with Se5Si10 (7.8 μmol g^−1^ FW) and Se10Si10 (7.9 μmol g^−1^ FW) demonstrating the greatest accumulation. SeNPs-10 (7.4 μmol g^−1^) also maintained elevated levels relative to the control (5.2 μmol g^−1^), establishing clear concentration–response relationships across treatments. Procyanidin concentration (Figure 5c) exhibited a comparable trend, with combined treatments Se_5_Si_10_ (28.6 g) and Se10Si10 (28.9 g) achieving maximum accumulation. Individual applications, SeNPs-5 (22.8 g) and SeNPs-10 (26.2 g), resulted in progressive increases above the control (18.5 g). Ascorbic acid content (Figure 5d) integrated these quality enhancement effects, as Se5Si10 (63.1 mg 100 g^−1^ FW) and Se10Si10 (60.7 mg 100 g^−1^ FW) achieved the highest vitamin C enrichment. SeNPs-10 (58.6 mg 100 g^−1^) maintained consistently elevated levels, while the control remained at baseline concentrations (45.2 mg 100 g^−1^).

Overall, the coordinated enhancement of flavonoid and quality-related metabolites demonstrates that combined Se-Si nanoparticle treatments effectively optimize fruit nutritional quality by activating flavonoid biosynthetic pathways and promoting antioxidant vitamin accumulation.

### 2.7. Physical Quality and Sugar Metabolism Enhancement

**Figure 6:**
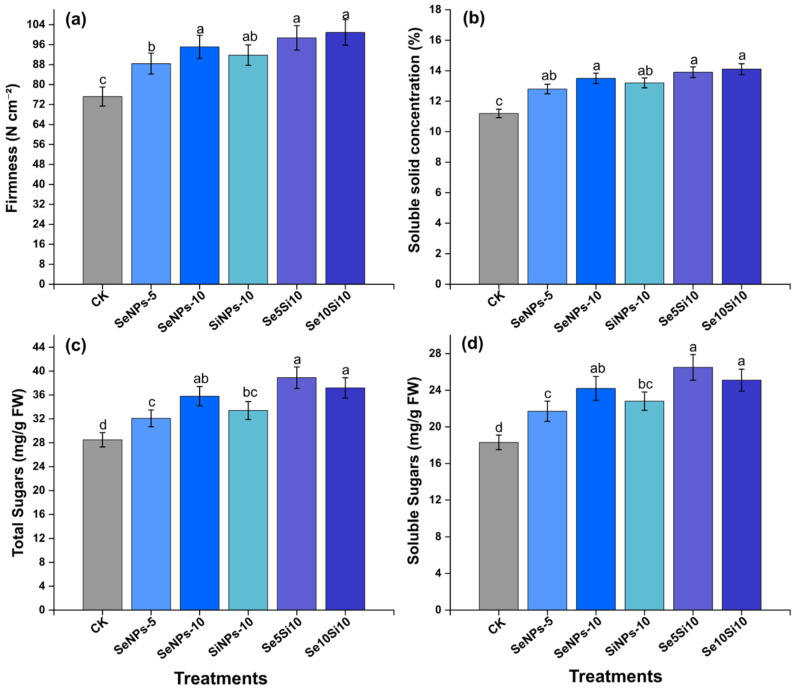
Effects of Se and Si nanoparticle treatments on physical quality and sugar metabolism in peach fruit. (**a**) Firmness, (**b**) Soluble solid concentration, (**c**) Total sugars, and (**d**) Soluble sugars content. Data represent means ± standard error of three biological replicates. Different letters indicate significant differences between treatments at *p* < 0.05.

Nanoparticle treatments significantly improved peach fruit physical quality attributes and sugar metabolism (Figure 6). Firmness enhancement (Figure 6a) exhibited substantial treatment effects with Se10Si10 achieving maximum fruit firmness (100.9 N cm^−2^), followed by Se5Si10 (98.7 N cm^−2^), representing 1.3-fold increases over control (75.2 N cm^−2^). Individual Se treatments SeNPs-5 (88.4 N cm^−2^) and SeNPs-10 (95.1 N cm^−2^) demonstrated progressive firmness enhancement, while SiNPs-10 (91.8 N cm^−2^) confirmed Si’s independent contribution to fruit texture improvement. Soluble solid concentration (Figure 6b) displayed coordinated enhancement patterns with combined treatments Se5Si10 (13.9%) and Se10Si10 (14.1%) achieving optimal sugar accumulation. SeNPs-10 (13.5%) maintained elevated soluble solids compared to control baseline (11.7%), establishing clear quality improvement under nanoparticle applications.

Total sugar content (Figure 6c) reinforced the metabolic enhancement effects with Se5Si10 (38.9 mg g^−1^ FW) and Se10Si10 (37.2 mg g^−1^ FW) demonstrating highest sugar accumulation. Individual treatments SeNPs-5 (32.1 mg g^−1^) and SeNPs-10 (35.8 mg g^−1^) achieved progressive increases above control levels (28.5 mg g^−1^). Soluble sugar concentration (Figure 6d) integrated the sugar metabolism optimization with Se5Si10 (26.5 mg g^−1^ FW) and Se10Si10 (25.1 mg g^−1^ FW) achieving maximum soluble sugar enhancement. SeNPs-10 (24.2 mg g^−1^) demonstrated substantial individual treatment efficacy, while control maintained lower sugar levels (18.7 mg g^−1^).

The integrated physical quality and sugar metabolism enhancement revealed that combined Se-Si nanoparticle treatments optimized fruit marketability through coordinated improvement of texture attributes and carbohydrate accumulation pathways.

### 2.8. Stress Indicators and Protein Metabolism

**Figure 7:**
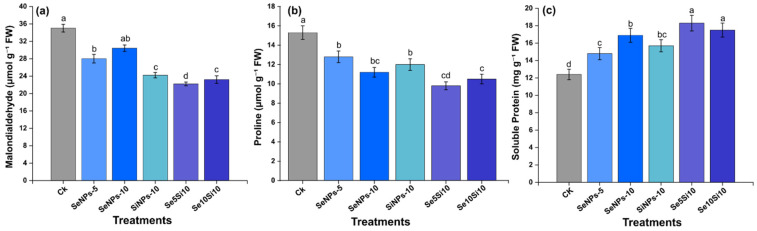
Effects of Se and Si nanoparticle treatments on oxidative stress markers and protein metabolism in peach fruit. (**a**) Malondialdehyde (MDA) content, (**b**) Proline content, and (**c**) Soluble protein content. Data represent means ± standard error of three biological replicates. Different letters indicate significant differences between treatments at *p* < 0.05.

Nanoparticle treatments significantly influenced stress-related metabolites and protein metabolism in peach fruits (Figure 7). MDA content (Figure 7a), a key indicator of lipid peroxidation, showed substantial treatment-dependent reductions. The combined treatments Se_5_Si_10_ (22.11 μmol g^−1^ FW) and Se_10_Si_10_ (23.21 μmol g^−1^ FW) achieved the lowest oxidative stress levels, representing approximately 37% decreases compared to the control (35.02 μmol g^−1^ FW). Individual Se applications, SeNPs-5 (28.02 μmol g^−1^) and SeNPs-10 (30.42 μmol g^−1^), resulted in moderate reductions, while SiNPs-10 (24.23 μmol g^−1^) highlighted Si’s independent antioxidant protection capacity.

Proline accumulation (Figure 7b) exhibited an inverse relationship with stress intensity, with the control showing the highest concentration (15.3 μmol g^−1^ FW) as a typical stress response. Combined treatments, Se_5_Si_10_ (9.8 μmol g^−1^) and Se_10_Si_10_ (10.5 μmol g^−1^), markedly reduced proline levels, indicating alleviated stress conditions. Individual Se treatments, SeNPs-5 (12.8 μmol g^−1^) and SeNPs-10 (11.2 μmol g^−1^), also demonstrated progressive stress mitigation effects. Soluble protein content (Figure 7c) further supported the metabolic enhancement pattern. Se_5_Si_10_ (18.3 mg g^−1^ FW) and Se_10_Si_10_ (17.5 mg g^−1^ FW) achieved the highest protein accumulation, while SeNPs-10 (16.9 mg g^−1^) maintained elevated protein synthesis relative to the control (12.4 mg g^−1^). These results indicate clear improvements in cellular metabolism under nanoparticle treatments.

Overall, the coordinated reduction in oxidative damage and enhancement of protein metabolism demonstrate that combined Se–Si nanoparticle treatments effectively optimize cellular homeostasis, thereby creating favorable conditions for improved fruit quality and stress resilience.

## 3. Discussion

The highest Se accumulation achieved through nanoparticle applications reflects enhanced cellular uptake and biotransformation mechanisms. The 95.2 nm spherical Se NPs demonstrated optimal size for foliar penetration through stomatal and cuticular pathways, consistent with recent findings indicating NPs < 100 nm exhibit highest bioavailability compared to bulk materials [25,26]. The commercially obtained Si NPs (24 nm), with their highly pure composition (95% Si) and uniform size distribution as confirmed by TEM and EDS analyses, possessed ideal physicochemical properties for agricultural applications [21]. The maintenance of fruit yield (52–56 kg/tree) across all treatments demonstrates that selenium and silicon nanoparticle applications at 5–10 mg/L concentrations operated within safe physiological thresholds without phytotoxic effects. This yield stability is crucial for commercial adoption, confirming that biofortification and quality enhancement can be achieved without compromising productivity, addressing a common concern in agricultural biofortification programs [27]. The significant increase in shoot silicon content (78% in SiNPs-10 treatment) confirms successful foliar uptake and translocation of Si nanoparticles, consistent with enhanced Si mobility reported in fruit crops [28]. The slightly reduced Si accumulation in combined Se-Si treatments (61–68% increase) suggests mild competitive effects during foliar absorption, possibly through shared cuticular penetration pathways [29]. This efficient Si translocation provides the physiological foundation for the observed enhancements in antioxidant activities and phenolic compounds, validating the dual nanoparticle approach for simultaneous biofortification and quality improvement. The Se size-dependent uptake occurs through endocytosis-mediated transport and plasmodesmatal movement, where smaller NPs exhibit reduced aggregation and enhanced dispersibility in plant tissues [16]. The preferential accumulation of organic Se forms (85% in SeNPs-10) suggests efficient biotransformation of inorganic Se NPs into selenoamino acids and selenoproteins through metabolic incorporation pathways involving selenocysteine and selenomethionine biosynthesis [1,30].

The biotransformation process involves sequential reduction of selenate to selenite, followed by assimilation into organic compounds through sulfur metabolism pathways. Se competes with sulfur for incorporation into amino acids, where selenocysteine synthase and methionine synthase facilitate the formation of selenoproteins essential for antioxidant function [31]. The higher organic Se percentage indicates active participation in protein synthesis rather than passive accumulation, suggesting metabolic integration that enhances bioavailability for human nutrition [32].

The dose-dependent Se biofortification pattern aligns with saturation kinetics of Se transport systems, where SeNPs-10 achieved optimal accumulation without toxicity symptoms. This supports the concept of hormetic responses in Se metabolism, where moderate concentrations enhance physiological functions while excessive levels trigger stress responses [33,34]. The hormetic curve reflects the dual nature of Se as both essential micronutrient and potential toxicant, with optimal benefits occurring within narrow concentration ranges. At cellular level, Se activates transcription factors like Nrf2 that regulate antioxidant gene expression, while excessive concentrations trigger oxidative stress through pro-oxidant mechanisms [35]. The molecular basis for Se tolerance involves glutathione-mediated detoxification pathways and compartmentalization of excess Se in vacuoles as less toxic organic forms. This protective mechanism explains why nanoparticle treatments maintained beneficial effects without exhibiting typical Se toxicity symptoms such as chlorosis or growth inhibition [12,36].

The synergistic enhancement observed with combined Se-Si treatments can be attributed to multiple coordinated molecular mechanisms. First, at the uptake level, silicon increases Se bioavailability through dual pathways: Si-mediated upregulation of SULTR transporters increases Se influx across leaf cell membranes, as Se competes with sulfate for the same transport systems [4,37]. Si modifies cell wall architecture by enhancing pectin methylesterase activity and increasing porosity, facilitating nanoparticle penetration and subsequent Se translocation [38]. Second, at the cellular level, Si optimizes the redox environment necessary for efficient Se metabolism. Si activates the ascorbate-glutathione cycle independently, providing enhanced reducing capacity that facilitates the conversion of selenate to selenite and subsequently to organic selenocompounds through the sulfur assimilation pathway [39].

Third, the observed synergistic enhancement of phenolic compounds involves transcriptional co-regulation. Both Se and Si activate WRKY and MYB transcription factors, which regulate phenylpropanoid biosynthesis genes including PAL, C4H, and 4CL [37,40]. However, their activation mechanisms differ: Se acts through mild oxidative signaling (controlled ROS generation), while Si functions through mechanical sensing and calcium signaling pathways [4]. The convergence of these distinct signaling forces on common target genes produces amplified transcriptional responses, explaining the superior phenolic accumulation in combined treatments compared to individual applications.

Si’s role in strengthening antioxidant defense systems created favorable conditions for Se metabolism and reduced oxidative stress associated with metal nanoparticle exposure [38]. The coordinated enhancement of antioxidant enzyme activities (POD: 2254 U g^−1^ in Se5Si10) reflects cross-talk between Se and Si signaling pathways. Si-mediated upregulation of antioxidant enzymes complements Se’s role in glutathione POD and selenoprotein synthesis, creating amplified cellular protection mechanisms [39,40].

This synergistic antioxidant enhancement involves multiple molecular mechanisms. Si activates the ascorbate-glutathione cycle through enhanced ascorbate POD and dehydroascorbate reductase activities, while Se functions as cofactor in glutathione POD and thioredoxin reductase systems [41]. The combined effect creates redundant antioxidant pathways that provide superior protection against reactive oxygen species generated during nanoparticle exposure and normal cellular metabolism [20]. Synergistic phenolic accumulation occurs through selenium-induced MYB/WRKY transcription factors activating phenylpropanoid genes (PAL, C4H, 4CL) while silicon enhances substrate availability via improved photosynthetic efficiency, creating amplified biosynthetic pathway expression [24].

The substantial enhancement of phenolic compounds and flavonoids through nanoparticle treatments indicates activation of stress-responsive secondary metabolite pathways. The 1.9-fold increase in chlorogenic acid accumulation with combined treatments reflects upregulation of phenylpropanoid biosynthesis under controlled oxidative stress conditions [42]. This enhancement occurs through Se-mediated activation of phenylalanine ammonia-lyase (PAL), the rate-limiting enzyme in phenylpropanoid biosynthesis, and subsequent upregulation of downstream enzymes including 4-coumarate: CoA ligase and cinnamate 4-hydroxylase [34]. The molecular mechanism involves Se-induced expression of MYB and bHLH transcription factors that regulate phenylpropanoid gene clusters. These transcription factors respond to Se-generated mild oxidative stress as signaling molecules, activating defense-related gene expression without triggering damage responses [38]. Si enhances this response by stabilizing enzyme conformations and providing structural support for metabolite accumulation in specialized storage cells.

Se’s role as a cofactor in antioxidant enzymes triggers compensatory responses in phenolic biosynthesis, while Si enhances structural stability of these compounds through cell wall reinforcement [39]. The preferential enhancement of specific flavonoids (rutin: 53.9 mg kg^−1^) suggests selective activation of flavonol biosynthetic branches. This specificity indicates that nanoparticle treatments modulate transcriptional regulation of key enzymes in flavonoid biosynthesis, particularly flavonol synthase and flavonoid 3′-hydroxylase [43,44].

The selective enhancement of rutin over other flavonoids reflects tissue-specific expression patterns of flavonoid biosynthetic enzymes and differential substrate affinities. Rutin biosynthesis involves quercetin 3-O-rutinosyltransferase, which exhibits enhanced activity under Se treatment due to improved cofactor availability and reduced oxidative enzyme damage [45]. This selective enhancement has important nutritional implications, as rutin possesses superior antioxidant properties and bioavailability compared to other flavonoids [12]. The improved physical quality parameters (firmness: 100.9 N cm^−2^, soluble solids: 14.1%) reflect comprehensive metabolic optimization rather than isolated effects. Enhanced sugar accumulation correlates with improved photosynthetic efficiency and carbon partitioning under optimized antioxidant conditions [45]. The coordination between firmness enhancement and sugar accumulation suggests nanoparticle treatments optimize cell wall metabolism and carbohydrate transport simultaneously.

The high rutin accumulation (53.9 mg kg^−1^) results from selenium-enhanced flavonol synthase (FLS) expression via ROS-mediated signaling, followed by glycosylation through UDP-glucose: flavonoid 3-O-glucosyltransferase to form the rutinoside moiety [46]. Elevated ascorbic acid content (63.1 mg/100 g) reflects enhanced GSH-dependent redox modulation through the ascorbate-glutathione cycle: selenium incorporation into glutathione peroxidase and glutathione reductase increases cycle efficiency, while silicon-enhanced NADPH availability drives ascorbate regeneration via DHAR and MDHAR enzymes [47].

The mechanistic basis for quality enhancement involves Se-mediated activation of sucrose phosphate synthase and sucrose synthase, key enzymes in sugar metabolism and transport. Se functions as cofactor in these enzymes while protecting them from oxidative inactivation, resulting in enhanced sugar synthesis and translocation to fruit tissues [27]. Si contributes by strengthening cell wall structure through enhanced pectin methylesterase activity and cellulose deposition, which improves fruit firmness while maintaining optimal porosity for metabolite transport [48]. The enhanced firmness also reflects improved calcium binding in cell walls, as Si facilitates calcium-pectin complex formation through ionic bridging mechanisms. This structural enhancement occurs without compromising fruit ripening processes, as evidenced by parallel increases in soluble solids and aromatic compound development [21]. The reduced oxidative stress markers (MDA: 22.11 μmol g^−1^) combined with enhanced protein synthesis indicates improved cellular homeostasis. This metabolic optimization creates favorable conditions for quality-related biosynthetic processes, explaining the coordinated enhancement across multiple quality parameters [49,50]. The reduction in lipid peroxidation reflects enhanced membrane stability through antioxidant protection and improved membrane composition via Se-dependent phospholipid metabolism [51].

Enhanced protein synthesis under nanoparticle treatments reflects improved ribosomal function and amino acid availability. Se enhances selenoprotein synthesis while protecting ribosomes from oxidative damage, while Si maintains optimal cellular ion balance necessary for protein folding and stability [52]. This coordinated protein enhancement supports enzyme activities throughout cellular metabolism, creating positive feedback loops that amplify quality improvements. The relationship between stress reduction and quality enhancement involves complex signaling networks where reduced oxidative stress allows energy reallocation from defense mechanisms to biosynthetic processes. This metabolic shift enables enhanced investment in secondary metabolite production, sugar accumulation, and structural improvements that define fruit quality [23].

The environmental persistence and food safety considerations of our Se-Si nanoparticle biofortification approach warrant attention for sustainable agricultural implementation. At our applied foliar concentrations (5–10 mg/L Se NPs, alone or combined with 10 mg/L Si NPs), approximately 85% of the Se was transformed into organic forms, which are nutritionally beneficial and environmentally benign. This efficient biotransformation aligns with recent findings by Wang et al. [16], who demonstrated through SP-ICP-MS analysis that foliar-applied Se NPs remain confined to leaf tissues, with Se accumulating in grain exclusively as ionic forms rather than nanoparticles, thereby eliminating risks of human exposure to nanomaterials through consumption. The synergistic application of Se-Si NPs at these low concentrations, combined with the controlled-release properties and rapid biotransformation to organic compounds, suggests minimal environmental persistence while ensuring food safety through the absence of nanoparticle residues in the harvested peach fruits.

These findings demonstrate that nanoparticle applications can achieve multiple agricultural objectives simultaneously: nutritional biofortification, quality enhancement, and stress mitigation. The synergistic effects of combined Se-Si treatments offer sustainable alternatives to conventional fertilization approaches, potentially reducing input requirements while maximizing nutritional and commercial value [9]. The efficiency of nanoparticle delivery systems reduces material waste and environmental impact compared to conventional fertilizers, while providing targeted nutrition that optimizes plant metabolism [53]. The preferential organic Se accumulation addresses consumer preferences for naturally occurring nutrient forms and supports the development of functional foods with enhanced health benefits. The coordinated quality improvements suggest potential for premium market positioning of biofortified fruits [2]. A public health perspective, the high organic Se content provides bioavailable nutrition that can contribute to addressing Se deficiency in populations with limited dietary diversity [54]. Additionally, nanoparticle concentrations can be optimized to achieve effective results while minimizing cost, making the treatment economically feasible and scalable for large-scale orchard production.

## 4. Materials and Methods

### 4.1. Experimental Site and Plant Material

The field experiment was conducted during the 2023 growing season at the Quzhou, Zhejiang Province, China (29°02′ N, 118°95′ E, altitude 150 m). The experimental site is characterized by a subtropical monsoon climate with an annual mean temperature of 17.3 °C, annual precipitation of 1650 mm, and 260 frost-free days. The study utilized 8-year-old peach trees (*Prunus persica* L.) with uniform growth vigor, planted at 4 × 5 m spacing in a commercial orchard.

The experimental soil belongs to typical red soil (Ultisol) commonly found in Zhejiang Province. Soil physicochemical properties at 0–20 cm depth are the following: pH 5.8 (acidic), organic matter 8.67 g kg^−1^, total nitrogen 1.24 g kg^−1^, available phosphorus 15.4 mg kg^−1^, available potassium 98.7 mg kg^−1^, exchangeable aluminum 2.8 cmol kg^−1^, and total Se 0.072 mg kg^−1^. The low Se content confirmed the Se-deficient nature of Quzhou red soils, which is characteristic of southeastern China’s acidic soils.

### 4.2. Nanoparticle Synthesis and Characterization

Se NPs were synthesized in the laboratory using a chemical reduction method [1]. Briefly, Se dioxide (SeO_2_) was dissolved in distilled water under constant magnetic stirring at room temperature. Sodium thiosulfate pentahydrate (Na_2_S_2_O_3_·5H_2_O, 156 mM) was used as the reducing agent and slowly added to the SeO_2_ solution. Sodium dodecyl sulfate (SDS, 0.01 M) was incorporated as a stabilizing agent to prevent nanoparticle aggregation. The reaction mixture was stirred continuously for 2 h at room temperature until a characteristic orange-red coloration appeared, indicating Se nanoparticle formation. The synthesized NPs were purified by centrifugation at 10,000 rpm for 15 min, washed three times with distilled water to remove excess reagents, and stored for subsequent analysis, while Si NPs (24 ± 4.3 nm) were purchased from Shanghai Macklin Biochemical Co., Ltd. (Shanghai, China).

Morphological characterization of Se and Si NPs were performed using field emission scanning electron microscopy (FE-SEM, Hitachi SU8010, Tokyo, Japan) operated at 5 kV. Elemental composition was analyzed using energy-dispersive X-ray spectroscopy (EDS) attached to the SEM system. Surface functional groups were identified by Fourier-transform infrared spectroscopy (FTIR, Thermo Fisher Scientific, Waltham, MA, USA) in the range of 400–4000 cm^−1^. Particle size distribution and zeta potential were determined using dynamic light scattering (DLS, Malvern Zetasizer Nano ZS, Malvern, UK).

### 4.3. Experimental Design and Treatment Application

The experiment was arranged in a randomized complete block design with six treatments and three replications. Each experimental plot consisted of three trees, with the central tree used for measurements to eliminate border effects. The treatments were as follows: CK (Control); foliar spray with distilled water, SeNPs-5; Se NPs at 5 mg L^−1^, SeNPs-10; Se NPs at 10 mg L^−1^, SiNPs-10; Si NPs at 10 mg L^−1^, Se5Si10; combined application of SeNPs (5 mg L^−1^) + SiNPs (10 mg L^−1^) and Se10Si10; combined application of SeNPs (10 mg L^−1^) + SiNPs (10 mg L^−1^).

Nanoparticle suspensions were prepared by dispersing the required amounts in distilled water containing 0.05% (*v*/*v*) Tween-80 as dispersing agent. The suspensions were ultrasonicated for 20 min before application to ensure homogeneous distribution and prevent particle aggregation. To maintain nanoparticle stability and avoid any oxidation-related alterations, Se nanoparticles were freshly synthesized before each foliar application. Three foliar applications were performed during critical phenological stages: (1) pre-bloom stage (10 days before flowering), (2) fruit setting stage (15 days after flowering), and (3) fruit development stage (60 days after flowering). Applications were conducted in early morning under calm weather conditions to minimize spray drift and maximize foliar absorption.

Each tree received approximately 3 L of spray solution applied using a battery-operated sprayer (16 L capacity) equipped with a cone nozzle, ensuring thorough coverage of both leaf surfaces and developing fruits.

### 4.4. Sample Collection and Preparation

Fruit harvest was conducted at commercial maturity stage, determined by fruit color development, flesh firmness, and soluble solid content. All fruits from three replicate trees per treatment were harvested and weighed using a digital scale (±0.01 kg precision). The number of fruits per tree was counted, and average fruit weight was calculated by dividing total yield by fruit number. Data were expressed as kg/tree and presented as means of three biological replicates, with each replicate consisting of three pooled trees (Table 1). Harvested fruits were immediately transported to the laboratory in ice-cooled containers within 1 h of collection.

For biochemical analyses, fruit samples were prepared by removing skin and stone, cutting flesh into small pieces, freezing in liquid nitrogen, and storing at −80 °C until analysis. Fresh fruit samples were used for physical quality measurements and Se content determination. All analytical determinations were performed in triplicate using fruits from different trees within each treatment.

### 4.5. Analytical Procedures

#### 4.5.1. Si Contents Analysis

Silicon content in shoots was determined using the alkaline digestion method of Manimaran et al. [20]. Dried samples (0.1 g) were digested with NaOH in an autoclave (121 °C, 20 min), neutralized, and Si concentration measured colorimetrically at 820 nm using the molybdenum blue method with sodium metasilicate standards.

#### 4.5.2. Se Content and Speciation Analysis

Total Se content was determined by inductively coupled plasma optical emission spectroscopy (ICP-OES, Optima 8300, PerkinElmer, Waltham, MA, USA) after microwave-assisted acid digestion. Fruit samples (2.0 g) were digested using concentrated HNO_3_:H_2_O_2_ (4:1 *v*/*v*) in a microwave digestion system (CEM Mars 6, Matthews, NC, USA) following EPA Method. Se speciation into organic and inorganic forms was performed using high-performance liquid chromatography coupled with hydride generation atomic fluorescence spectrometry (HPLC-HG-AFS). Protein extraction was performed using Tris-HCl buffer, followed by enzymatic digestion with protease XIV to release Se-containing amino acids. To access Se bioaccessibility, peach fruit (3 g) was mixed with 10 mL gastric buffer (pH 1.8) and 0.8 mL pepsin solution, then incubated at 37 °C for 90 min. The pH was adjusted to 6.5 using NaHCO_3_, followed by addition of 2 mL pancreatin-bile solution and further incubation for 90 min at pH 7.0. Samples were centrifuged at 4000 rpm for 15 min at 4 °C. The supernatant was filtered and analyzed for Se by AFS [4].

$$Bioaccesibility\;\left(\%\right)=[\frac{\left(Se\;Conc.\right)\;vitro\;digestion\;sol.\times \;Vol.\left(µg\right)total\;vol\;in-vitro\;digestion\;sol.\;\;}{\left(Se\;Conc.\right)\;peach\times Weight\;of\;\;peach\;\left(µg\right)}]\times 100$$

#### 4.5.3. Antioxidant Enzyme Activity Assays

Fresh peach (2.0 g) was homogenized in 10 mL of ice-cold 50 mM phosphate buffer (pH 7.0) containing 1 mM EDTA, 2% (*w*/*v*) polyvinylpolypyrrolidone (PVP), and 0.1% (*v*/*v*) Triton X-100. The homogenate was centrifuged at 15,000× *g* for 20 min at 4 °C, and the supernatant was used for enzyme assays [52]. All enzyme activities were determined using commercial detection kits from Suzhou Koming Biotechnology Co., Ltd. (Suzhou, China).

Peroxidase (POD) activity was determined using the guaiacol colorimetric method [55]. The reaction mixture consisted of enzyme extract, guaiacol solution, and H_2_O_2_, with absorbance measured at 470 nm. Superoxide dismutase (SOD) activity was assessed using the nitroblue tetrazolium (NBT) inhibition method at 560 nm. Catalase (CAT) activity was measured by monitoring H_2_O_2_ decomposition at 240 nm. Total antioxidant capacity (TAC) was determined using the 1,1-diphenyl-2-picrylhydrazyl (DPPH) radical scavenging assay.

#### 4.5.4. Phenolic Compound Analysis

Individual phenolic compounds were analyzed following the method described by Li et al. [56] with modifications. Fresh fruit (100 mg) was ground under liquid nitrogen and extracted with 1 mL of 60% ethanol through ultrasonication for 30 min at 30 °C, followed by centrifugation at 12,000 rpm for 2 min. The extraction process was repeated twice, and the combined extracts were purified using 100 mg C18 solid phase extraction in 2 mL centrifuge tubes. Total phenolic content was quantified using the Folin–Ciocalteu reagent method with gallic acid as standard. Individual phenolic acids (chlorogenic acid, caffeic acid, ferulic acid) were analyzed by reverse-phase HPLC equipped with an HPLC reversed-phase C18 column (Athena C18-WP 2.1 × 50 mm, 3 μm). The mobile phase consisted of acetonitrile and 0.1% formic acid in water using gradient elution.

#### 4.5.5. Flavonoid and Quality Component Analysis

Total flavonoid content was determined using the aluminum chloride colorimetric method with quercetin as standard [57]. Rutin content was quantified by HPLC analysis using authentic standards. Ascorbic acid content was measured using commercial assay kits (Suzhou Koming Biotechnology Co., Ltd., Suzhou, China) following the manufacturer’s protocols. Soluble sugar content was determined using the anthrone colorimetric method. Total sugar content was analyzed using the 3,5-dinitrosalicylic acid (DNS) method. Soluble protein content was quantified using the Coomassie Brilliant Blue G-250 staining technique with bovine serum albumin as standard.

#### 4.5.6. Physical Quality Parameters

Fruit firmness was measured using a digital penetrometer (GY-4, Shanghai, China) with an 11.1 mm diameter probe, penetrating to 10 mm depth at two equatorial positions per fruit. Soluble solid content (SSC) was determined using a digital refractometer (PAL-1, Atago, Tokyo, Japan) calibrated with distilled water at 20 °C [58].

#### 4.5.7. Stress-Related Metabolites

Malondialdehyde (MDA) content was measured using the thiobarbituric acid reactive substances (TBARS) method with commercial detection kits. Proline content was determined using the acid ninhydrin method with L-proline as standard. The measurements were conducted following standard protocols adapted from Moselhy et al. [59].

### 4.6. Statistical Analysis

Statistical analyses were performed using SPSS 26.0 software at a 95% confidence level, employing one-way ANOVA followed by Duncan’s multiple range test (*p* < 0.05). Data are presented as means ± standard error of three biological replicates, with each replicate consisting of measurements from different trees within each treatment block. Graphs were generated using Origin Pro 2025 software.

## 5. Conclusions

This study successfully demonstrated the efficacy of combined Se and Si nanoparticle treatments for achieving dual objectives of nutritional biofortification and fruit quality enhancement in peach production. SeNPs-10 achieved remarkable Se biofortification with 5.4-fold increases in fruit Se content (0.47 mg kg^−1^) and 85% organic Se accumulation, addressing critical Se deficiency challenges in Se-poor regions. Combined Se-Si treatments demonstrated highest synergistic effects, with Se5Si10 and Se10Si10 optimally enhancing the antioxidant enzyme activation (POD: 2254 U g^−1^), phenolic compounds (chlorogenic acid: 267 mg kg^−1^), and physical quality parameters (firmness: 100.9 N cm^−2^, soluble solids: 14.1%). The coordinated enhancement of multiple quality parameters reflects comprehensive metabolic optimization rather than isolated effects. Stress mitigation through reduced oxidative damage (MDA: 22.11 μmol g^−1^) indicates improved cellular homeostasis supporting quality development. These findings provide practical protocols for sustainable production of nutritionally improved fruits that address both consumer preferences and public health nutrition objectives. They also indicate environmentally responsible alternatives to conventional fertilization leading to significant biofortification outcomes applicable to diverse horticultural crops. 

### Limitations

The study includes single-season data collection and a small sample size, which may affect the broader applicability of our conclusions despite consistent trends across parameters. Multi-year trials with larger sample sizes across varying environmental conditions are necessary to confirm the reproducibility and commercial viability of the studied nanoparticle applications.

## Figures and Tables

**Table 1:** Fruit yield parameters and shoot silicon content in peach trees following foliar application of Se and Si nanoparticles. Data are means ± SD (*n* = 3). Different letters indicate significant differences (*p* < 0.05).

Parameters	CK	SeNPs-5	SeNPs-10	SiNPs-10	Se5Si10	Se10Si10
Fruit yield (kg/tree)	52.4 ± 3.8 ^a^	53.6 ± 4.1 ^a^	54.2 ± 3.9 ^a^	55.8 ± 4.3 ^a^	55.1 ± 4.5 ^a^	54.7 ± 4.0 ^a^
No. of fruits/tree	312 ± 25 ^a^	318 ± 28 ^a^	324 ± 26 ^a^	335 ± 29 ^a^	328 ± 27 ^a^	326 ± 24 ^a^
Average fruit weight (g)	168 ± 12 ^a^	169 ± 11 ^a^	167 ± 13 ^a^	167 ± 11 ^a^	168 ± 12 ^a^	168 ± 13 ^a^
Si in shoots (mg g^−1^ DW)	0.82 ± 0.06 ^c^	0.85 ± 0.05 ^c^	0.84 ± 0.07 ^c^	1.46 ± 0.09 ^a^	1.38 ± 0.08 ^a^^b^	1.32 ± 0.07 ^b^

## Data Availability

The data presented in this study are available on request from the corresponding author. (The data are not publicly available due to privacy or ethical restrictions.)
